# Transportation of the Wounded

**Published:** 1918-09

**Authors:** 


### TRANSPORTATION OF THE WOUNDED

The difficulties of transporting wounded soldiers from battle areas constitute one of the most serious problems with which an army has to deal. In the symposium of the Research Society on "Transportation of the Wounded" various phases of the problem were discussed in a way that will be helpful to the American army in developing its ambulance train transport service.

Colonel Gallie, who has had charge of the ambulance train service of the British Medical Corps, opened the discussion by a description of the development of the British hospital trains from only four primitive units in 1914 to forty-one trains, equipped as hospitals, with a total capacity of 25000 beds. Colonel Gallie designed the railway ambulance service for the British service, and the magnificent hospital trains that are now in use by the American army were made according to his design, and manufactured in England. He thinks it important that hospital trains be kept moving instead of waiting for a full load in the beginning of a battle. He outlined the methods employed in the British army in caring for the wounded on the hospital trains. Colonel Gallie also discussed barge transportation where there are available waterways. He thought it an ideal method of caring for severely wounded patients because there is no jarring or motion; but the barges should be thoroughly equipped, and each one should be in charge of a medical officer capable of giving wounded men thorough hospital treatment. He also dealt briefly with ambulance ships which are used in transporting soldiers to England.

Colonel Ensor discussed the evacuation of wounded men from forward areas, and said that in some sections of the British army horse-drawn ambulance wagons are used to convey wounded from the firing line to divisional dressing stations; and from there they are moved to the casualty clearing hospitals by means of G. S. wagons and motor "lorries". He thought it important that the main dressing stations should be situated on roads which the general supply wagons use in going to and fro from the trains to the re-filling points, because instead of returning empty they may be utilized to transport wounded soldiers.

Colonel Poe of the British Medical Service spoke of the advantages of the motor ambulance convoy to an army corps, and stressed the importance of a continuous "flow of motor cars" so that there would be no delay at any point. He called attention to the heating of cars by the extension of the exhaust, the pipes from which are carried along in front of each seat; but care must be used for proper ventilation of the ambulance in order that the wounded should not be in danger of asphyxiation. Cars should always carry a sufficient number of hot water bottles and blankets to keep the patients warm.

Lt-Col. Thompson said that three important points should be stressed : first, distance; second, speed; third, the welfare of the patient during the journey. He thought the longer journeys were compensated for by placing wounded soldiers out of range and sound of the battle, in a surgical center where their wounds may be immediately and thoroughly attended to, particularly those with abdominal and chest wounds. He said that it could not be too strongly impressed upon motor ambulance chauffeurs that the greatest care and skill should be used in driving; and that in chest wounds, particularly, rough driving has been known to cause fatal hemorrhage.

Colonel Poe gave some general directions as to the preparation of the wounded for the trip, and for their care en route to the clearing station, or base hospital. He also dealt with conditions requiring special consideration, such as fracture, hemorrhage, etc.

Médecin-Major Proust, representing the French army, said that the ideal method ot transporting the wounded man, once examination is begun, was uninterrupted transport to the place where his wounds could be looked after promptey and properly. He described the methods in use in the French army which he thought had been quite satisfactory.

Colonel Percy Jones, Chief of the American Ambulance Service with the French Armies, discussed the "Evacuation of Wounded by Motor Vehicles in the Rear Section of the

Advance Zone ". He said that no system will meet the needs under all conditions, as every evacuation on an unusually large scale from a battle area must be a rule unto itself. He thought it important to standardize ambulances, and said that he considered the Ford ambulance capable of going anywhere that a vehicle drawn by animals could go; that it was light; easy to run and maintain; and required very little road space. His tribute to the Ford car would make good campaign literature for " Henry ", in his race for United States Senate.

Colonel Jones insisted that time is the chief element in getting the wounded man to the point where his wounds can be looked after promptly. He said that this could be best managed by the organization of various zones for this purpose, and he gave the details of the plan that is in practical operation in the American Ambulance Service with the French armies. He thought that with any system sufficient elasticity should be provided for keeping in constant touch with the units served. He said that the ambulance driver should understand map reading, and should be thoroughly familiar with the roads and topography in the advance zone in which he is expected to operate.

The resume of the discussions before the Research Society on " Transportation of the Wounded ", as published in this number of *War Medicine*, makes a valuable contribution to military surgery at a time when the American army is making its share of the sacrifice necessary to crush German militarism.

